# Pilot Study on the Relationship Between Different Lower Limb Raising Velocities and Trunk Muscle Contraction in Active Straight Leg Raise

**DOI:** 10.3390/jfmk9040276

**Published:** 2024-12-18

**Authors:** Kohei Yoshikawa, Noriyuki Kida, Takumi Jiroumaru, Yuta Murata, Shinichi Noguchi

**Affiliations:** 1 Kanazawa Orthopaedic and Sports Medicine Clinic, 881 Ono, Ritto 520-3016, Japan; kohei.yoshikawa.601520@gmail.com; 2 Graduate School of Science and Technology, Kyoto Institute of Technology, Kyoto 606-8585, Japan; 3 Faculty of Arts and Sciences, Kyoto Institute of Technology, Kyoto 606-8585, Japan; 4 Department of Physical Therapy, School of Health Science, Bukkyo University, Kyoto 604-8418, Japan; t-jiromaru@bukkyo-u.ac.jp; 5 Furu Clinic, 1098 Konancho Terasho, Koka 520-3301, Japan; 6 Department of Physical Therapy, School of Rehabilitation, Biwako Professional University of Rehabilitation, Higashiomi 527-0145, Japan; s-noguchi@pt-si.aino.ac.jp

**Keywords:** active straight leg raise, lower limb raising velocities, surface electromyography

## Abstract

Background/Objectives: The active straight leg raise requires intricate coordination between the hip, knee, pelvis, and spine. Despite its complexity, limited research has explored the relationship between lower limb raising velocity and trunk muscle motor control during an active straight leg raise in healthy individuals. This study aimed to explore the potential effects of increased lower limb raising velocity on core muscle contractions during active straight leg raises. Methods: Six healthy adult men (mean age: 24.5 ± 2.5 years) participated in this study. Electromyography signals were recorded using surface electrodes placed on the rectus abdominis, external oblique, and internal oblique/transverse abdominis muscles. The participants performed active straight leg raises at three different velocities: 3 s, 2 s, and as fast as possible (max). The electromyography data were analyzed from 250 ms before to 1000 ms after movement initiation, with muscle activity expressed as a percentage of the maximal voluntary isometric contraction. Statistical analyses were conducted using non-parametric tests, including the Friedman test for overall differences, followed by pairwise Wilcoxon signed-rank tests with Bonferroni correction for multiple comparisons (*p* < 0.05). Results: During the 250 ms before movement initiation, the internal oblique/transverse abdominis, external oblique, and rectus abdominis muscles showed greater activity in the max condition compared to the 3 s and 2 s conditions (Friedman test, *p* < 0.05), but no significant differences were found in pairwise comparisons (Wilcoxon test, *p* > 0.05). Similarly, during the 500 ms after movement initiation, internal oblique/transverse abdominis activity was higher in the max condition, with no significant pairwise differences observed. Conclusions: Faster lower limb raising velocities during active straight leg raise may enhance core stability by activating anticipatory and sustained internal oblique/transverse abdominis, external oblique, and rectus abdominis activity on the raised limb side. Training to promote this activation could improve dynamic stability in rapid or asymmetric movements.

## 1. Introduction

Core stability plays a crucial role in athletic performance, daily movements, and injury prevention; however, its definition remains subject to diverse interpretations. Core stability has no absolute definition because it comprises various interacting factors [1]. Generally, it is categorized into “static stability” and “dynamic stability.” Static stability is supported by structural components such as bones, joints, and ligaments [2], whereas dynamic stability is controlled by muscles and the nervous system [3,4]. Dynamic stability involves the coordination of passive elements such as bones and ligaments, active elements such as muscles and tendons, and neural elements to maintain spinal stability, allowing safe movement [5]. In sports contexts, core stability is essential for the efficient transmission and control of force and movement to the extremities. In sports, core stability is defined as the ability to control the position and movement of the trunk over the pelvis, allowing for optimal generation, transfer, and control of forces and movements to the extremities [5]. In addition, core stability requires adaptive control during movement in response to different situations [1]. Although core stability in sports has no unified definition, this study defines dynamic core stability as a state in which passive, active, and neural elements work together to maintain movement within a certain range.

The core typically refers to the muscles of the abdomen, spinal muscles, diaphragm, pelvic floor muscles, and the muscles surrounding the hips, which together form a cylindrical structure supporting the spine. Core muscles play a critical role in maintaining posture and transferring force. Deep muscles, such as the transverse abdominis (TrA), internal obliques (IO), and multifidus (MF), are considered more suitable than superficial muscles for controlling the segmental movements of the spine [6]. These deep muscles coordinate with superficial muscles depending on the velocity and type of movement [7]. For example, deep muscles are reportedly activated before movements in tasks such as single-leg standing and the active straight leg raise (ASLR) test [8,9,10]. Although deep muscles finely tune the segmental spinal stability, superficial muscles stabilize the entire spine through kinetic chains.

Several studies have demonstrated the impact of movement velocity on core muscle activity. During shoulder flexion movements in a standing position, faster movement velocities promote greater anticipatory muscle activity of the TrA [11]. TrA activation has also been observed during hip flexion and extension, suggesting that movement velocity influences core muscle activity [12]. Anticipatory contraction of the TrA during rapid limb movements is critical for maintaining core stability. Thus, core muscle function may vary depending on movement velocity and posture.

Various methods have been used to assess core stability. Static assessments such as the plank and side-bridge tests are commonly used to evaluate core muscle endurance and strength [13,14,15]. However, these tests do not reflect the stability or muscle coordination during dynamic movements. Methods for evaluating dynamic stability include the Sherman core stability test, which assesses core stability during movement by evaluating the response of core muscles to external forces and movements, as well as balance abilities [16,17]. The star excursion balance test (SEBT) is used to assess the dynamic stability of the core and lower limbs in single-leg standing by measuring the reach in multiple directions [17,18,19,20]. Furthermore, the functional movement screen (FMS) is used to evaluate basic movement patterns, assessing overall stability, flexibility, and coordination [21,22,23]. Although the FMS and SEBT involve whole-body movements, they have limitations in directly assessing the core muscles. The ASLR test, which evaluates the coordination of the lower limbs and core muscles while raising one leg in the supine position, is effective for assessing dynamic core stability [24,25].

The ASLR is a standard test for assessing the coordination between the lower limb and core muscles by flexing the hip while lying supine with the knee extended. This test can effectively measure dynamic core stability [26]. The ASLR is well suited for electromyographic (EMG) analysis, allowing the evaluation of muscle onset time and contraction intensity, which provides a more detailed understanding of dynamic core stability. Studies on healthy individuals have reported that the IO and TrA are activated during the later stages of ASLR, whereas the external oblique (EO) and rectus abdominis (RA) muscles are delayed compared with the psoas major and IO muscles when the leg is lifted [27]. In patients with lumbopelvic pain, TrA and IO activation during ASLR was lower than that in pain-free groups [28]. Although these studies have indicated that muscle activity during ASLR contributes to core stability and postural control, the effect of movement velocity on muscle activity remains unclear. Different movement velocities may significantly affect the timing and intensity of muscle contraction. Faster movements require faster muscle contractions and greater explosive force, whereas slower movements require sustained muscle activity. Understanding the relationship between movement velocity and muscle activity is crucial [29]. Even for the same movement, the effect of movement velocity on the activity patterns, timing, and intensity of core muscles remains unclear. Further research is needed, particularly on how the deep and superficial core muscles work together to ensure stability in response to different movement velocities during tasks such as ASLR.

It is generally accepted that faster movements prompt anticipatory TrA activation to maintain dynamic core stability. This pilot study aimed to explore the effects of increased lower limb raising velocity during ASLR on the contraction of core muscles, particularly the IO/TrA, EO, and RA, to provide preliminary data and a methodological foundation for future comprehensive studies.

## 2. Materials and Methods

### 2.1. Participants

Six healthy adult men (24.5 ± 2.5 years, 170.5 ± 5.1 cm, 68.2 ± 9.0 kg) participated in this experiment. Participants with a history of pain, significant postural abnormalities, or serious neurological or respiratory conditions were excluded. Informed consent was obtained from all the participants. This study was approved by the Ethics Committee of Kanazawa Orthopedic Surgery Clinic (Kanazawa-OSMC-2024-003) and conducted in accordance with the Declaration of Helsinki.

### 2.2. Electromyography Recording

The skin was carefully prepared by shaving excess hair and reducing the skin impedance to below 5 kΩ. After drying the skin, pairs of Ag/AgCl surface electrodes (SMP-300, METS Co., LTD., Tokyo, Japan) with a size of 19 mm per side were placed 20 mm apart at the following locations: rectus abdominis (RA), 1 cm above and 2 cm lateral to the umbilicus [30]; external oblique (EO), 1 cm above the horizontal line passing through the umbilicus and 1 cm lateral to the RA boundary [31]; and IO/TrA, 1 cm medial to the anterior superior iliac spine (ASIS) and 0.5 cm below the line connecting the ASIS [32]. This placement captures the combined activity of the IO and TrA muscles, as demonstrated in a previous study. Additionally, the muscle fibers of the IO/TrA were identified under ultrasound guidance, and it was confirmed that there was no overlap with the EO. The electrodes were placed bilaterally at each site for a total of six locations. To minimize cross-talk, careful attention was paid to electrode placement, inter-electrode distance, and orientation along the muscle fibers [33]. EMG signals were sampled at 1000 Hz, amplified, and collected using an EMG analysis software (VitalRecorder2 ver. 3.8.4. 1403, KISSEI COMTEC Co., Ltd., Nagano, Japan).

### 2.3. Kinematics

Kinematic data were collected by attaching a foot sensor to the heel, which defined the movement initiation point.

### 2.4. Experimental Task

**Figure 1:**
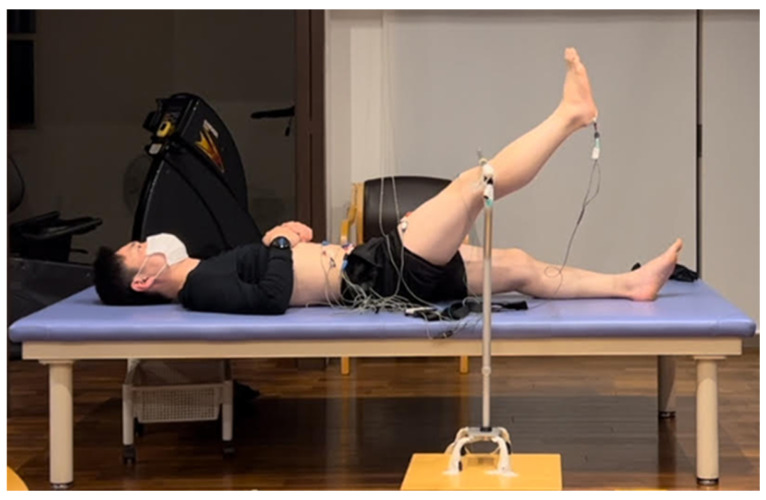
Experimental design of the active straight leg raise task.

EMG and kinematic data were synchronously recorded during ASLR. The participants performed the ASLR task in the supine position with their legs straight, raising their right lower limb to 45° of hip flexion at three different velocities. A bar was set up such that the participant’s patella would touch it at 45° of hip flexion (Figure 1).

The three conditions were grouped based on the speed of raising the leg to 45°. In the 3 s group (3 s) (slow; angular velocity ω = 15°/s), the leg was raised at a slow speed, taking exactly 3 s. In the 2 s group (2 s) (moderate; ω = 22.5°/s), the leg was raised at a moderate speed, taking 2 s. Finally, in the max group (max) (as fast as possible; ω = approximately 45°/s), the leg was raised at the fastest possible speed, typically in about 1 s. Each condition was performed six times with the order of the trials randomized, and the movements were initiated following verbal commands. The participants performed MVICs for 5 s in the supine against manual resistance for each muscle. Two MVIC trials were performed for each muscle with sufficient rest between trials, and the higher value from the two trials was used.

### 2.5. Data Analysis

EMG data were analyzed based on kinematic data using movement initiation as the reference point. The analysis focused on the period from 250 ms before movement initiation to 1000 ms after initiation. First, the raw data were bandpass-filtered at 20–500 Hz and then full-wave rectified. The root mean square of the EMG amplitude was calculated using a 50 ms window for each ASLR trial and MVIC.

For the data 250 ms before movement initiation (pre-250 ms), the average was calculated using a 50 ms window. The data 1000 ms after movement initiation were divided into four intervals as follows: 0–250 ms (post-250 ms), 251–500 ms (post-500 ms), 501–750 ms (post-750 ms), and 751–1000 ms (post-1000 ms). The average value for each 50 ms interval was calculated. Muscle activity levels are expressed as a percentage of MVIC (%MVIC) (MATLAB R2024a, MathWorks, Inc., Natick, MA, USA).

### 2.6. Statistical Analysis

Due to the small sample size and the results of the normality tests, non-parametric statistical methods were employed. The Friedman test was used to assess differences among the three velocities (3 s, 2 s, and max). When the Friedman test indicated significant differences, post-hoc pairwise comparisons were conducted using the Wilcoxon signed-rank test with Bonferroni correction to adjust for multiple comparisons. Statistical significance was set at *p* < 0.05. All statistical analyses were performed using the SPSS ver. 29.0.2 (IBM Corp., Armonk, NY, USA).

## 3. Results

During the 250 ms before movement initiation, the IO/TrA, EO, and RA muscles on the same side as the raised lower limb tended to be higher compared to the 3 s and 2 s conditions, as indicated by the Friedman test. For the IO/TrA, the activity in the max condition (7.5 ± 4.8%) tended to be higher compared to the 3 s (3.2 ± 1.8%) and 2 s (3.2 ± 1.8%) conditions (χ^2^ = 9.3, *p* = 0.009, Kendall’s W = 0.78). For EO, the activity in the max condition (18.5 ± 3.6%) tended to be higher compared to the 3 s (10.9 ± 3.9%) and 2 s (11.2 ± 2.7%) conditions (χ^2^ = 7.0, *p* = 0.030, Kendall’s W = 0.58). For RA, the activity in the max condition (6.6 ± 3.3) tended to be higher compared to the 3 s (3.6 ± 1.1%) and 2 s (3.8 ± 1.1%) conditions (χ^2^ = 8.3, *p* = 0.016, Kendall’s W = 0.69) (Table 1).

However, subsequent pairwise comparisons using the Wilcoxon test did not reveal significant differences between any of the conditions (*p* > 0.05 for all comparisons) (Table 2, Table 3 and Table 4).

During the 500 ms after movement initiation, the IO/TrA tended to be higher in the max condition compared to the 3 s and 2 s conditions, as indicated by the Friedman test (χ^2^ = 9.0, *p* = 0.011, Kendall’s W = 0.75). For the IO/TrA, the activity in the max condition (8.8 ± 7.0%) tended to be higher compared to the 3 s (4.0 ± 2.3%) and 2 s (4.2 ± 2.6%) conditions. However, subsequent pairwise comparisons using the Wilcoxon test did not reveal significant differences between any of the conditions during this period (*p* > 0.05 for all comparisons) (Table 5).

## 4. Discussion

This pilot study aimed to investigate how differences in lower limb raising velocities during ASLR influence the contraction of core muscles, particularly the IO/TrA, EO, and RA. During the 250 ms before movement initiation, the muscle activities of the IO/TrA, EO, and RA on the same side as the raised lower limb tended to be higher compared to the 3 s and 2 s conditions, as indicated by the Friedman test. Similarly, during the 500 ms after movement initiation, the IO/TrA also tended to be higher compared to the 3 s and 2 s conditions. However, pairwise comparisons using the Wilcoxon test did not reveal significant differences between conditions during either period.

These findings suggest that both anticipatory and early post-movement contractions of the IO/TrA, EO, and RA may play a role in maintaining core stability during fast lower limb raising in the supine position.

Unlike previous studies that investigated postural control during task performance in standing positions [7,8,10], this study examined postural control in the supine position. When standing, gravity exerts a greater effect, requiring more active stabilization by the core muscles [11]. In the supine position, core stability is partially supported by passive elements, such as bones and ligaments, reducing the demand for strong core muscle contractions [3]. However, as movement velocity increases, maintaining core stability requires anticipatory contractions of both deep muscles, such as the IO/TrA, and superficial muscles, including the EO and RA. During the max condition, the coordinated contraction of the EO, RA, and IO/TrA on the same side as the raised lower limb is essential for ensuring core stability, whereas, during slower movements (2 s and 3 s conditions), anticipatory muscle contractions may not be as necessary, allowing stability to be maintained more easily. The notable finding that the IO/TrA, EO, and RA on the same side as the raised lower limb exhibited increased activity before movement initiation aligns with the concept of “feedforward postural control”, where core muscles activate in anticipation of limb movement to ensure core stability [34]. This result highlights the importance of preparatory muscle activation across both deep and superficial muscles, particularly during rapid movements like those observed in the max condition, to counteract the increased demands for core stabilization.

The findings partially align with previous studies on movements in the standing position. For instance, during fast shoulder flexion in standing, anticipatory contraction of the deep muscle TrA is promoted regardless of the direction of preparatory trunk movement, and superficial muscles like the IO and EO exhibit direction-specific activity. However, in standing, the activity of deep muscles (TrA and MF) is more critical for trunk support [11].

Similarly, our study revealed that, in the relatively stable supine position, the deep muscle IO/TrA on the same side as the raised lower limb showed a trend toward greater activation, along with a tendency for higher activity in the superficial muscles EO and RA on the same side during fast movements. This suggests that both deep and superficial core muscles are actively involved in maintaining core stability during fast lower limb raising in the supine position. These results further support the notion that posture significantly influences muscle activity patterns and that the muscles involved in maintaining core stability adapt depending on the posture.

The unilateral dominance of the IO/TrA, EO, and RA on the side of the raised lower limb can be explained as an adaptive response of the core muscles to asymmetric movements. Previous studies have reported that unilateral limb movements result in asymmetric activity of the core muscles to ensure stability on that side [5]. This finding aligns with the increased activity of the IO/TrA, EO, and RA on the lower limb raising side in this study, suggesting that unilateral core stability contributes to lower limb movement control.

The effect of movement velocity on core muscle activity tended to be evident, with the EO and RA showing greater activity in the fast velocity condition. Additionally, the deep muscle IO/TrA also exhibited a trend toward greater activation in the fast condition. Faster movements generate greater rotational forces (moments) around the hip joint, requiring both superficial muscles like the EO and RA and deep muscles such as the IO/TrA to contract more rapidly to maintain core stability. As movement velocity increases, the moment at which movement initiation increases and the activities of the EO, RA, and IO/TrA increase accordingly to counteract these forces. This suggests that both superficial and deep core muscles are actively involved in stabilizing the core during fast movements.

Deep and superficial muscles play different roles in core stability. Deep muscles like the TrA, IO, and MF contribute to segmental stabilization of the spine, whereas superficial muscles like the EO, RA, and IO support overall spinal stabilization [34,35]. Previous studies have reported a muscle-activation pattern during dynamic movements in which deep muscles activate first, followed by superficial muscles [34]. In this study, conducted in a relatively stable supine position, it was observed that the deep muscle IO/TrA showed a trend toward higher activity before movement initiation. Similarly, the superficial muscles EO and RA also demonstrated a trend toward higher activity before movement initiation during fast movements. This coordinated activation of deep and superficial muscles suggests that these muscle groups work together to maintain core stability before and during fast lower-limb raising in the supine position. These findings highlight the influence of posture on muscle activity and suggest that the muscles involved in maintaining core stability adapt their activation patterns according to the posture. Previous studies have focused on movements performed in standing positions, where trunk stabilization is critical [12]. In contrast, ASLR involves lower limb raising in a supine position with a relatively light load, suggesting that the demands on core stability are lower. However, even under light loads, the IO/TrA, EO, and RA on the side of the raised lower limb may contribute to overall movement stability by controlling trunk rotation and lateral flexion during lower limb raising.

Furthermore, the IO/TrA showed a trend toward higher activity even during the 500 ms after movement initiation. This suggests that the IO/TrA plays a crucial role in maintaining core stability not only before but also during movement. On the other hand, the EO and RA were primarily active before movement initiation, indicating that their role may be limited to ensuring core stability before movement. These findings suggest that deep and superficial muscles perform distinct roles and function at different times in maintaining core stability, with the IO/TrA playing a central role in sustaining core stability throughout the movement.

Additionally, the lack of differences in EO and RA activity after movement initiation suggests that, under the maximal velocity condition, significant muscle exertion to counteract a large moment at movement initiation was followed by the leg being raised by inertia. Consequently, the need for muscle exertion decreased after movement initiation. In ASLR with a light load, the EO and RA are most active before or at the onset of movement, after which the movement requires only the maintenance of muscle strength. This indicates that high muscle activity was not necessary after movement initiation, allowing for stable motion.

The results of this study provide valuable insights into core training in sports and rehabilitation. Eliciting the coordinated activity of both deep and superficial core muscles, such as the IO/TrA, EO, and RA, on the same side as the raised lower limb before movement initiation may play a crucial role in enhancing dynamic core stability. This coordinated muscle activation is particularly important during fast movements, as it helps stabilize the core in anticipation of increased demands. For athletes, training programs that focus on improving the anticipatory and cooperative activation of these core muscles could be essential in activities that require rapid and explosive movements.

This study had several limitations. The small sample size and lack of statistical power limit the ability to detect significant differences, potentially leading to false negatives. However, as a pilot study, these findings hold significant value in laying the groundwork for hypothesis-driven experimental research. The insights gained from this exploratory study are critical for designing future experiments with refined methodologies and larger, more diverse sample populations. Moreover, the results are specific to the studied population and conditions, which may limit their generalizability. Future research is required to validate these findings, expand on the observed trends, and provide robust evidence to confirm the role of lower limb raising velocity in enhancing core muscle activity and stability. Additionally, the results are specific to the studied population and conditions, which may limit their generalizability. Future research is required to validate these findings with a larger sample size, more diverse populations, and more refined methodologies to confirm the observed trends and provide robust evidence. In this study, we assessed muscle activity of the IO/TrA and EO using surface electromyography. We acknowledge that recording sEMG signals from deep muscles like the TrA has inherent limitations, and it is difficult to completely eliminate the influence of crosstalk [35]. However, we took measures to minimize these effects through careful electrode placement. These limitations should be considered when interpreting the results.

## 5. Conclusions

This study provides preliminary evidence suggesting that the coordinated activity of both deep and superficial core muscles, including the IO/TrA, EO, and RA, plays crucial roles in maintaining core stability during high-velocity lower limb raising in ASLR. The IO/TrA exhibited activity both before and during movement, suggesting its continuous contribution to core stabilization across different phases of the movement. Meanwhile, the EO and RA were primarily active before movement initiation, highlighting their anticipatory roles in ensuring core stability. Practically, incorporating training programs that promote both the anticipatory activation of the IO/TrA, EO, and RA and the sustained activation of the IO/TrA during lower limb raising or unilateral movements in the supine position may be beneficial for enhancing dynamic core stability. Strengthening these muscles could improve performance in sports and daily activities, particularly in movements that require rapid or asymmetric core stabilization.

## Figures and Tables

**Table 1:** EMG (%MVIC) values for each muscle, with the mean ± SD, median (IQR), and Friedman test results across trials.

		3 s		2 s		Max		Friedman Test			
		Mean ± SD	Median (IQR)	Mean ± SD	Median (IQR)	Mean ± SD	Median (IQR)	χ^2^	df	*p*-Value	Kendall’s W
Right IO/TrA	pre-250 ms	3.2 ± 1.8	3.0 (1.6–5.1)	3.2 ± 1.8	3.1 (1.6–5.1)	7.5 ± 4.8	5.4 (4.7–10.8)	9.3	2	0.009 *	0.78
	post-250 ms	3.8 ± 2.3	3.7 (1.8–6.0)	3.9 ± 2.4	3.8 (1.8–6.0)	8.4 ± 5.8	7.4 (4.8–11.2)	4.0	2	0.135	0.33
	post-500 ms	4.0 ± 2.3	4.0 (1.9–6.0)	4.2 ± 2.6	3.9 (1.9–6.4)	8.8 ± 7.0	6.9 (4.6–11.7)	9.0	2	0.011 *	0.75
	post-750 ms	4.2 ± 2.5	4.1 (2.0–6.3)	5.2 ± 3.2	5.1 (2.1–7.8)	6.4 ± 4.1	5.4 (3.4–9.8)	2.3	2	0.311	0.31
	post-1000 ms	4.6 ± 2.7	4.5 (2.1–6.9)	5.7 ± 3.9	5.2 (2.3–8.7)	5.1 ± 2.7	4.9 (2.4–8.1)	3.0	2	0.223	0.25
Left IO/TrA	pre-250 ms	2.8 ± 1.3	2.9 (1.5–4.1)	2.8 ± 1.3	2.9 (1.4–4.0)	4.6 ± 2.3	4.9 (2.3–6.9)	5.3	2	0.069	0.44
	post-250 ms	3.2 ± 1.7	3.6 (1.5–4.7)	3.3 ± 1.9	3.4 (1.4–4.9)	8.0 ± 5.7	6.6 (3.8–14.4)	4.3	2	0.115	0.36
	post-500 ms	3.1 ± 1.5	3.5 (1.5–4.3)	3.0 ± 1.6	3.4 (1.5–4.4)	6.6 ± 4.5	5.6 (3.5–10.7)	1.0	2	0.607	0.83
	post-750 ms	3.3 ± 1.6	3.8 (1.6–4.5)	3.2 ± 1.6	3.6 (1.5–4.6)	6.1 ± 3.9	5.3 (3.5–9.8)	1.3	2	0.513	0.11
	post-1000 ms	3.4 ± 1.6	4.0 (1.7–4.7)	3.3 ± 1.7	3.9 (1.6–4.7)	5.4 ± 3.1	5.2 (3.5–8.3)	0.3	2	0.846	0.28
Right EO	pre-250 ms	10.9 ± 3.9	10.0 (7.8–14.1)	11.2 ± 2.7	10.9 (9.6–13.1)	18.5 ± 3.6	19.4 (15.9–21.4)	7.0	2	0.030 *	0.58
	post-250 ms	10.7 ± 3.7	9.9 (7.7–13.1)	10.8 ± 2.0	11.3 (8.6–12.6)	15.4 ± 7.4	15.2 (9.8–20.2)	2.3	2	0.311	0.19
	post-500 ms	10.8 ± 3.8	10.3 (7.7–13.6)	11.0 ± 1.8	10.9 (9.6–12.8)	17.0 ± 8.2	14.0 (10.2–26.6)	2.3	2	0.311	0.19
	post-750 ms	11.1 ± 3.7	10.9 (7.9–13.7)	11.4 ± 2.3	10.7 (9.6–14.1)	16.0 ± 6.6	14.1 (11.8–23.7)	4.0	2	0.135	0.33
	post-1000 ms	12.7 ± 5.0	12.3 (8.3–16.8)	11.8 ± 3.1	11.3 (8.9–14.4)	14.2 ± 5.7	12.6 (10.5–16.8)	0.3	2	0.846	0.03
Left EO	pre-250 ms	7.1 ± 4.1	5.8 (4.7–8.6)	6.5 ± 1.4	6.3 (5.2–7.5)	8.7 ± 2.8	8.6 (6.7–11.5)	2.3	2	0.311	0.19
	post-250 ms	7.5 ± 3.3	6.6 (4.8–10.7)	6.8 ± 2.6	7.3 (4.6–8.7)	10.5 ± 6.4	9.3 (5.2–16.4)	1.3	2	0.513	0.11
	post-500 ms	6.7 ± 3.1	6.2 (4.4–8.5)	6.6 ± 2.2	6.8 (4.8–8.8)	10.0 ± 4.5	6.8 (4.8–8.8)	4.0	2	0.135	0.33
	post-750 ms	6.9 ± 3.5	6.1 (4.6–8.8)	6.9 ± 2.5	6.8 (5.1–8.4)	8.8 ± 3.1	9.4 (5.3–10.8)	3.0	2	0.223	0.25
	post-1000 ms	7.1 ± 3.2	6.7 (4.9–8.6)	7.5 ± 2.1	7.1 (5.6–9.1)	7.8 ± 2.8	6.8 (5.8–10.1)	1.0	2	0.607	0.08
Right RA	pre-250 ms	3.6 ± 1.1	4.1 (2.5–4.5)	3.8 ± 1.1	4.0 (2.8–4.8)	6.6 ± 3.3	6.4 (4.4–8.9)	8.3	2	0.016 *	0.69
	post-250 ms	3.9 ± 1.1	4.3 (3.0–4.7)	4.1 ± 1.4	4.3 (2.8–5.2)	5.0 ± 2.6	5.3 (2.6–7.1)	3.0	2	0.223	0.25
	post-500 ms	3.7 ± 1.0	3.8 (3.0–4.7)	4.1 ± 1.2	4.5 (2.8–5.0)	4.9 ± 2.9	4.9 (2.1–7.0)	2.3	2	0.311	0.19
	post-750 ms	3.7 ± 1.0	3.9 (2.9–4.5)	4.3 ± 1.6	4.4 (2.9–5.4)	4.7 ± 2.1	5.1 (2.9–6.5)	4.0	2	0.135	0.33
	post-1000 ms	3.9 ± 1.1	4.0 (3.3–4.7)	4.5 ± 1.7	4.3 (3.4–5.7)	4.2 ± 2.2	4.5 (2.0–5.9)	1.3	2	0.513	0.11
Left RA	pre-250 ms	3.8 ± 1.6	3.7 (2.0–5.6)	3.9 ± 1.4	3.8 (2.8–5.5)	5.0 ± 2.1	5.1 (3.2–7.1)	4.3	2	0.115	0.36
	post-250 ms	3.8 ± 1.5	3.7 (2.3–5.4)	3.8 ± 1.3	3.5 (2.9–5.1)	4.3 ± 2.2	3.8 (2.8–6.3)	1.0	2	0.607	0.08
	post-500 ms	3.6 ± 1.6	3.4 (2.1–5.3)	3.6 ± 1.2	3.2 (2.8–4.7)	4.3 ± 1.9	4.3 (2.7–6.0)	1.3	2	0.513	0.11
	post-750 ms	3.8 ± 1.4	4.3 (2.2–4.8)	3.7 ± 1.3	3.5 (2.5–4.9)	4.4 ± 1.7	4.4 (3.2–5.9)	1.3	2	0.513	0.11
	post-1000 ms	4.2 ± 1.8	4.4 (2.1–5.8)	3.8 ± 1.3	3.8 (2.5–5.0)	3.9 ± 1.8	4.3 (2.0–5.3)	2.3	2	0.311	0.19

*, *p* < 0.05. EMG, electromyography; %MVIC, percentage of maximal voluntary isometric contraction; SD, Standard Deviation; IQR, Interquartile Range; IO/TrA, internal oblique/transverse abdominis muscles; EO, external oblique; RA rectus abdominis.

**Table 2:** Results of the Wilcoxon test following the Friedman test for right IO/TrA in pre-250 ms.

	z-Value	*p*-Value (Adjusted)	Effect Size (r)
3 s vs. 2 s	0.314	1	0.128
3 s vs. max	−2.201	0.084	0.899
2 s vs. max	2.201	0.084	0.899

**Table 3:** Results of the Wilcoxon test following the Friedman test for right EO in pre-250 ms.

	z-Value	*p*-Value (Adjusted)	Effect Size (r)
3 s vs. 2 s	−0.105	1	0.043
3 s vs. max	−1.992	0.138	0.813
2 s vs. max	−2.201	0.084	0.899

**Table 4:** Results of the Wilcoxon test following the Friedman test for RA in pre-250 ms.

	z-Value	*p*-Value (Adjusted)	Effect Size (r)
3 s vs. 2 s	0.943	1	0.385
3 s vs. max	2.201	0.084	0.899
2 s vs. max	−1.992	0.138	0.813

**Table 5:** Results of the Wilcoxon test following the Friedman test for right IO/TrA in post-500 ms.

	z-Value	*p*-Value (Adjusted)	Effect Size (r)
3 s vs. 2 s	0.524	1	0.214
3 s vs. max	2.201	0.084	0.899
2 s vs. max	−2.201	0.084	0.899

## Data Availability

The original contributions presented in the study are included in the article, further inquiries can be directed to the corresponding author.
